# Changing case Order to Optimise patterns of Performance in mammography Screening (CO-OPS): study protocol for a randomized controlled trial

**DOI:** 10.1186/1745-6215-15-17

**Published:** 2014-01-10

**Authors:** Sian Taylor-Phillips, Matthew G Wallis, Helen Parsons, Janet Dunn, Nigel Stallard, Helen Campbell, Sarah Sellars, Ala Szczepura, Simon Gates, Aileen Clarke

**Affiliations:** 1 Warwick Medical School, The University of Warwick, Coventry CV4 7AL, UK; 2 Cambridge Breast Unit and NIHR Cambridge Biomedical Research Centre, Box 97, Cambridge University Hospitals NHS Foundation Trust, Biomedical Campus, Hills Road, Cambridge CB2 0QQ, UK; 3 Health Economics Research Centre, Nuffield Department of Population Health, University of Oxford, Old Road Campus, Headington, Oxford OX3 7LF, UK; 4 NHS Cancer Screening Programmes, Public Health England, Fulwood House, Old Fulwood Road, Sheffield S10 3TH, UK

**Keywords:** Breast cancer, Fatigue, Mammography, Observer performance, Screening, Vigilance decrement

## Abstract

**Background:**

X-ray mammography remains the predominant test for screening for breast cancer, with the aim of reducing breast cancer mortality. In the English NHS Breast Screening Programme each woman’s mammograms are examined separately by two expert readers. The two readers read each batch in the same order and each indicates if there should be recall for further tests. This is a highly skilled, pressurised, repetitive and frequently intellectually unchallenging activity where readers examine one or more batches of 30–50 women’s mammograms in each session. A vigilance decrement or performance decrease over time has been observed in similar repetitive visual tasks such as radar operation.

**Methods/Design:**

The CO-OPS study is a pragmatic, multi-centre, two-arm, double blind cluster randomised controlled trial of a computer software intervention designed to reduce the effects of a vigilance decrement in breast cancer screening. The unit of randomisation is the batch. Intervention batches will be examined in the opposite order by the two readers (one forwards, one backwards). Control batches will be read in the same order as one another, as is current standard practice. The hypothesis is that cancer detection rates will be higher in the intervention group because each readers’ peak performance will occur when examining different women’s mammograms. The trial will take place in 44 English breast screening centres for 1 year and 4 months. The primary outcome is cancer detection rate, which will be extracted from computer records after 1 year of the trial. The secondary outcomes include rate of disagreement between readers (a more statistically powerful surrogate for cancer detection rate), recall rate, positive predictive value, and interval cancer rate (cancers found between screening rounds which will be measured three years after the end of the trial).

**Discussion:**

This is the first trial of an intervention to ameliorate a vigilance decrement in breast cancer screening.

**Trial registration:**

ISRCTN46603370 (submitted: 24 October 2012, date of registration: 26 March 2013).

## Background

### Epidemiology and burden of breast cancer

Breast cancer is a leading cause of mortality in women and was the cause of death for 10,280 women in England and Wales in 2010. It was responsible for 12% of all deaths of women in their 50s and 8% of deaths of women in their 60s [1].

In England, up to 2.3 million women aged 47 to 73 years are projected to have breast cancer screening each year [2,3]. The recall rate is 40 per 1,000 women screened, and the cancer detection rate is 7.8 per 1,000 women screened; therefore, 32 healthy women per 1,000 screened are recalled for further tests (false-positive recalls) [3].

Currently, 66% of all breast cancers in women 50 to 64 years of age in England are detected by screening [3]. This occurs because some women choose not to be screened and the screening test is not perfect. Cancers diagnosed between screens (interval cancers) either grow *de novo* between screening rounds or are not seen or picked up by the programme. As with all screening programmes, the National Health Service Breast Screening Programme (NHSBSP) aims to keep interval cancers to a minimum, with a target rate of 2.3 per 1,000 women [4,5]. However, these are difficult targets; the rates achieved are closer to 3 per 1,000 women [6].

When investigating methods of improving cancer detection, one has to be mindful of the potential for increasing overdiagnosis and overtreatment (that is, cancers detected at screening and treated which never would have presented or caused problems within the patient’s life). It is important to ensure that any additional test or new methodology that increases cancer detection has a matched fall in interval cancer rates.

### Existing knowledge

In the NHSBSP, each woman’s mammograms are examined separately by two specialists, each of whom indicates whether there should be a recall for further tests. These experts are radiologists, advanced radiography practitioners or breast clinicians, (henceforth referred to for simplicity as *readers*). If the two readers disagree, the case is referred for arbitration, usually by a third reader or a group of readers. For workflow reasons, 30 to 50 women’s mammograms are grouped together into a batch and are typically read without a break. In a mammography reading session, a reader may examine one, two or more batches. On average, readers assess two women’s mammograms per minute [7]. This is a highly skilled, pressurised and repetitive, but frequently intellectually nonchallenging, activity. A vigilance decrement manifesting as a performance decrease over time has been observed in a wide range of similar repetitive visual tasks such as radar operation [8].

Current practice is for both readers to examine each batch of mammograms in the same order as one another; thus any vigilance decrement will occur when reading the same woman’s mammograms. We hypothesise that presenting batches to the first and second readers in a different order from one another may increase the sensitivity of screening. A trial is required to determine whether such improvements would be realised in practice.

## Methods/Design

Our hypothesis is that if the second reader examines the batch of mammograms in the order opposite that of the first reader, the overall cancer detection rates will increase. This will happen through increasing rates of disagreement between readers because each reader’s peak performance will occur when examining different cases.

The trial will be carried out in accordance with Good Clinical Practice (GCP) as detailed in the Medical Research Council GCP guidelines [9] and the following protocol. The trial will be reported in line with the CONSORT (Consolidated Standards of Reporting Trials) statement [10] and the further guidance available for cluster randomised controlled trials [11]. Ethical approval was granted by the Coventry and Warwickshire National Health Service (NHS) Research Ethics Committee on 27 June 2012 (Reference 12/WM/0182). Formal approval was also granted by the Research and Development office of each NHS Trust involved in the study.

### Trial summary

The trial is a pragmatic, multicentre, two-arm, double-blind, cluster randomised controlled trial of a computer software intervention designed to reduce the effects of a vigilance decrement in breast cancer screening. The intervention changes the order in which the readers examine the batches of mammograms. The centres will be NHSBSP centres with digital mammography equipment. A cluster for randomisation comprises a batch of mammograms (approximately 30 to 50 women’s mammograms). Intervention batches will be examined in the opposite order by the two readers (one in appointment order and one in reverse appointment order). Control batches will be read in the same order as one another as is current standard practice. The unit for analysis is the individual woman’s outcome.

Standard practice is for both readers to read the batch of mammograms in the order in which the women were booked in for their appointments. In the control group, both readers will read each batch in the same order as one another, but this may be in appointment order or in reverse appointment order (↓↓ or ↑↑). In the intervention group, both readers will read the mammograms in the order opposite one another (↓↑ or ↑↓). This design blinds the readers because if they read in the reverse order, they will not know if they are reading the intervention or control group. The study software which will be used to run the trial will be embedded in the National Breast Screening Service (NBSS) software system in every English breast screening centre as part of routine updating of the NBSS system.

The pilot study will be conducted in three screening centres for 2 weeks to ascertain practical issues with implementation and to test the data collection tool, which will extract the results from the screening centres’ electronic database (NBSS). Concurrently, a survey of all English breast screening centres will be conducted to ascertain key characteristics relevant to the trial which may differ between centres, such as the method of arbitration used. These details will be described in the report and considered in the statistical analysis of the trial to ascertain whether the effectiveness of the intervention is affected by these characteristics.

The trial will last 16 months in 44 centres and will include all women attending routine digital mammography screening during the study period. The data gathered during the first 12 months (1.1 million women) will be derived from the NBSS database to determine whether the intervention has increased the cancer detection rate. These data will include all items for the primary and secondary analyses, with the exception of interval cancer rate. The interval cancer data will be collected 3 years after the end of the study period to ascertain whether there has a been a decrease in the rate of interval cancers and therefore whether the intervention reduced the number of cancers missed at screening.

### Trial aims and outcomes

The primary aim of the trial is to determine whether presenting the cases to the first and second readers in the order opposite one another increases the cancer detection rate (true-positive rate). The secondary aims and outcome measures of the trial are as follows:

1. Is there a difference in the number of disagreements between readers in each study arm? This is the proposed mechanism by which an increase in cancer detection rate occurs and therefore acts as a surrogate outcome, particularly if the effect size is smaller than anticipated or recruitment targets are not met. The rate of disagreement refers to the proportion of cases for which one reader thought the case should be recalled and the other reader thought the case should not be recalled.

2. Is there a difference in 3-year interval cancer rate in each study arm? Furthermore, as the interval cancer rate can be considered an approximation of the false-negative rate, is this change (if any) concurrent with changes in the cancer detection rate between study arms (primary outcome)? The interval cancer rate is measured as the rate of cancers detected symptomatically in the 3 years subsequent to screening and therefore will be collected 3 years after the trial implementation is complete.

3. Do the recall rate and positive predictive value (PPV) differ between the study arms?

4. With regard to NHS considerations, is the intervention cost-effective when compared with the control arm? The lifetime cost-effectiveness of the intervention will be modelled using the primary outcome from the trial. Are there any effects introduced by the trial methodology? Specifically, are the cancer detection and recall rates affected by introducing reverse reading order? The cancer detection rate and the recall rate for both versions of the control arm (↓↓ and ↑↑) will be compared to measure any adverse effects of reading in reverse order.

### Statistical considerations

#### Primary analysis

To determine whether the cancer detection rate is higher in the intervention group than in the control group, a two-tailed analysis will be conducted using a multilevel logistic regression model. Levels in the model will be case, batch and centre. To prevent overfitting, each level will be included in the final model only if it explains a sufficient portion of the variability and improves model fit. The intervention may be more effective in certain subgroups, particularly younger women, women whose cases are read at the very beginning or end of the batch and the first batches to be examined in a workday. These subgroups will be analysed separately as specified in the statistical analysis plan and agreed by the Trial Steering Committee.

Analysis will be conducted as intention to treat, with all cases randomised included in the analysis. Missing data through loss to follow-up will occur in both groups for women who are recalled from screening but either did not attend the follow-up appointment or for whom no records exist in the database concerning the results of that appointment. Multiple imputation and sensitivity analyses to examine the effects of any missing data on the model will be considered if appropriate.

#### Secondary analyses

To determine whether the disagreement rate between readers, interval cancer rate and recall rate are different between the intervention and control groups (secondary aims 1, 2 and 3), the same methods as described for the primary analysis above will be used. The PPV of cancer detection in each study arm will be calculated as the proportion of women recalled who are found to have cancer (secondary aim 3).

The effects of the trial introducing the reverse reading order (secondary aim 5) will also be analysed. The recall and cancer detection rates for the two reading groups which make up the control arm will be compared (↓↓ compared to ↑↑). Models will be constructed in the same way as the primary analysis, but using reading order, not trial group membership, as the predictor of cancer detection rate and recall rate.

To generate secondary outcome 4 (estimates of cost-effectiveness), the primary outcome from the trial will be used as an input into a health economic model of breast cancer screening. This model will be developed by expanding an earlier breast cancer model described by Campbell *et al*. [12] and will predict lifetime costs and effects for both the intervention and control arms. The model simulates the underlying breast cancer disease process according to prognosis upon presentation and includes adjuvant therapies, the long-term risk of recurrence (both local and metastatic) and treatment and long-term survival following recurrence. It will be used to model the changes in costs, life-years and quality-adjusted life years brought about by a potential change in the cancer detection rate.

### Power and sample size

Currently, 14,700 cancers are detected by screening each year, and the cancer detection rate is 7.8 per 1,000 women screened [3]. Detection of one extra cancer case per 2,000 women screened would increase the cancer detection rate to around 8.3 cancers per 1,000 women screened. To detect this change of 0.5 cancers per 1,000 women using a two-tailed test at 80% power and 5% significance, 501,361 women are required in each arm.

As randomisation occurs at the batch level, however, collected data are clustered and this must also be taken into consideration. The sample size of a clustered study must be increased by the design effect (DE), which is calculated as *DE* = 1 + (*m* - 1)*ρ* for a given intraclass correlation coefficient (ICC; *ρ*) and cluster size (*m*). The ICC was calculated using previous data in a logistic binomial Gaussian model (method B) with 1,000 Monte Carlo simulations [13]. Hence, using the ICC of 0.002 and a cluster (batch) size of 40 women produces a DE of 1.09, increasing the sample size required to 546,890 in each arm.

**Figure 1:**
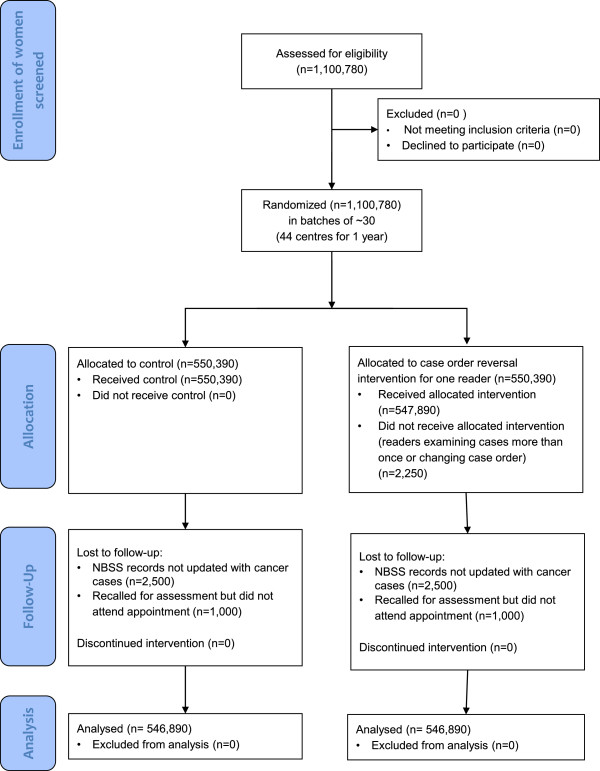
**Planned baseline CONSORT 2010 flow diagram for cancer detection analysis.** All batches of women attending routine digital mammography screening at participating centres during the trial will be randomised to either the intervention or control group. Follow-up data may not be available for a small proportion of women who either do not attend a recall for assessment or for whom records are not updated at the point of data collection. NBSS, National Breast Screening Service.

There is no adjustment for crossover, because once the intervention is applied to a screening centre, each batch will automatically be randomly assigned to the intervention or control group by the NBSS computer system and the intervention will be applied automatically by the same system. A woman could be lost to follow-up if her NBSS records have not been updated or if she is recalled from screening for further tests and does not attend her follow-up appointment. This is uncommon, however, so we have assumed low dropout rates of 3,500 women in each arm, inflating the sample required to 550,390 in each arm. Therefore, as detailed in Figure 1, the overall sample size required is for 1,100,780 women, or 44 breast screening centres for 1 year (On the basis that in England there are 82 centres each screening around 25,000 women per year).

### Recruitment

There are 80 breast screening centres in England. About 44 breast screening centres will be recruited into the trial (depending on centre size), which will last for 1 year, 4 months. After the first year of the trial (allowing 2 months for follow-up appointments to determine which women have cancer), the data for 1 year of screening at 44 centres will be downloaded and analysis of the primary outcome of cancer detection rate and the secondary outcomes will be completed. Analysis of missed cancer rates will be undertaken at 3 years after completion of the administration of the intervention.

There will be no extra time commitment for NHS staff in administering the trial at each centre, as the design is fully computerised and changes to batch reading orders will be automatically downloaded using each centre’s latest software update. Importantly, this scheme is anticipated to help with reaching recruitment targets.

### Eligibility

To be included, screening centres must use double-reading of screening mammograms and must have at least one piece of digital mammography equipment used for screening. All women who undergo mammography screening using digital equipment during the study period at the study centres will be included.

Centres which use single reading of mammograms, centres that have no digital mammography equipment used for screening (for example, the software intervention does not work in the old equipment) and women who attend symptomatic breast clinics will be excluded.

### Postrandomisation withdrawals and exclusions

Centres participating may withdraw from the trial at any time without prejudice. Upon withdrawal, or at any time before the data is analysed, centres can choose whether their data is collected and used in the analysis. Initial survey results describing centre characteristics will be used to analyse patterns of withdrawals from the trial, unless the centre requests that their data not be used in this way.

### Compliance and contamination

Compliance is expected to be very high because, after centre-level consent is given, all implementation is automated. In addition, deviation from the protocol would be very difficult. There is no method available to move cases between intervention and control groups. Case order can be changed manually by selecting the ‘ignore’ option rather than inputting a screening result, so that either the mammogram can be reviewed at a later stage or a result can be input and revised later. These events will change the reading order and produce some level of contamination. However, analysis will be based on intended order of reading. Data will also be collected on actual reading order to measure levels of contamination and their effects on outcomes. These effects are expected to be extremely small (on the order of 1 per 500 cases) because screening is a fast-moving, high-volume activity, and there is little time in practice to change reading order or to come back to cases at a later stage. Full data management procedures can be found in the statistical analysis plan.

### Consent

Informed consent will be obtained at the centre level from the director of the service, who is also usually the lead reader. Consent will be obtained at the centre level rather than by patient or reader because it is at this level that the intervention is applied. In addition, the intervention can be considered an alternative form of standard practice because no aspect of the way in which the mammograms are reviewed or evaluated is altered other than the reading order.

Directors of breast screening centres will be contacted in the first instance by email introducing the study with a copy of the participant information sheet and informed consent form attached. They will then receive a follow-up telephone call 1 week later. Each centre director will be offered a copy of the research protocol and the Integrated Research Application System ethics form and a visit from one member of the investigation team to introduce the study if desired. The study software embedded in the NBSS software will remain inactive until a signed consent form is received from the centre and local research and development approvals are granted, at which point it can be activated simply and quickly. The study can commence in each centre by simply changing the software settings to activate the intervention.

In the unlikely event that information becomes available which may be relevant to the participant’s willingness to continue participating in the trial (for example, findings from other research studies), then the directors of every participating breast screening centre will be informed immediately by email and follow-up telephone call.

### Recruitment and randomisation

The rate of accrual will be monitored at the centre level. If it falls appreciably below the projected level, the reasons will be identified and remedial actions will be taken to protect the power of the trial and alleviate concerns about selective entry and other aspects of quality. Randomisation will be automatically computer-generated at the point at which the batch is ready to be read on the NBSS system. Only batches of cases to be read as part of the NHS breast screening programme will be randomised. Cases from symptomatic clinics will be excluded prior to randomisation. Randomisation will be carried out with equal probability to one of four groups: control (both read in appointment order: ↓↓), control (both read in reverse appointment order: ↑↑), intervention (first reader in appointment order and second reader in reverse appointment order: ↓↑) and intervention (first reader in reverse appointment order and second reader in appointment order ↑↓).

### Blinding

The women screened will not be aware of whether their mammograms are read as part of an intervention or a control batch. The readers will not know the arm of the trial to which each batch has been assigned, because batches will be read in appointment order and reverse appointment order in both arms of the trial.

## Discussion

The experimental design is between subjects, so readers will not be burdened with reading cases twice (once in each experimental condition). This design has lower statistical power than a within-participant approach, however, so recruitment targets are high to achieve 80% power. Sensitivity analysis for the estimate of effect size indicates that it is very sensitive to small changes in effect size. If the effect size is as low as 200 extra cancers detected per year in England, it may still be considered clinically significant, but overall sample size would need to be 14 million women to detect such an effect. Achieving a sample of this size would be unrealistic; therefore, there is a risk of the trial being underpowered. However, an effect size of 200 extra cancers detected in England each year would be detectable in the form of a surrogate for cancer detection rate, which is the rate of disagreements between readers. This parameter is included as a secondary outcome. If recruitment targets are not met, then the rate of disagreements, as a surrogate for cancer detection rate, may have to be used as the primary outcome.

One possible mechanism for harm from the intervention is associated with reading the cases in reverse appointment order, which is not currently standard practice. To blind the readers to group allocation, however, reading in reverse appointment order is present equally in the intervention and control groups, so a two-tailed test would not detect this harm. Therefore, a secondary analysis of recall rate and cancer detection rate of cases read in appointment order and reverse appointment order will be carried out to determine whether reading a batch in reverse appointment order affects performance.

This study is a pragmatic multicentre, two-arm, double-blind, cluster randomised controlled trial of a computer software intervention designed to reduce the effects of a vigilance decrement in breast cancer screening. The intervention and randomisation are applied automatically using computer software, thus reducing the cost of running the trial and limiting the possibility of allocation and treatment bias. This study design also reduces the administrative burden on participating centres, thus removing one of the barriers to participation. The trial is double-blinded because neither the women screened nor the readers will be aware of whether each batch is in the intervention or control group.

### Trial status

The trial has National Health Service ethical approval, and the software has been activated in 45 centres in the United Kingdom. Data collection is due to start in February 2014.

### Trial steering committee

The fellowship advisory group also acts as the trial steering committee. There is no need for a data-monitoring committee, as there will be no interim data available to monitor.

## Competing interests

The authors declare that they have no competing interests.

## Authors’ contributions

SP conceived the study, secured the funding, led the design process and drafted the paper. HP performed the sample size calculations. HP and NS advised on the statistical analysis plan. MW, SS and AC contributed to securing the funding and to designing and implementing the study. HC contributed to study design and implementation, specialising in its health economics elements. AS, JD and SG contributed to study design. All authors helped redraft the manuscript and read and approved the final version for publication.
